# Predictors of quality of life among inpatients in forensic mental health: implications for occupational therapists

**DOI:** 10.1186/s12888-018-1605-2

**Published:** 2018-01-19

**Authors:** Padraic O’ Flynn, Roisin O’ Regan, Ken O’ Reilly, Harry G Kennedy

**Affiliations:** 1 National Forensic Mental Health Service, Central Mental Hospital, Dundrum, Dublin, Ireland; 2 Department of Psychiatry, Trinity College, Dublin, Ireland

## Abstract

**Background:**

Optimising quality of life (QOL) for service users in a forensic hospital is an important treatment objective. The factors which contribute to QOL in this setting are currently unclear. The aim of this study was to analyse the predictors of QOL amongst service users within an inpatient forensic mental health hospital.

**Methods:**

This study is a naturalistic, cross-sectional, observational study. Fifty-two male service users with schizophrenia or schizoaffective disorder participated in the study. QOL was measured using the World Health Organisation QOL Bref. We used the Engagement in Meaningful Activity Survey (EMAS), ward atmosphere was measured using the Essen Climate Evaluation Schema (EssenCES), occupational functioning was assessed using the Social and Occupational Functioning Scale (SOFAS). We also collected level of ward security, length of stay and community leave data.

**Results:**

Stepwise regression showed that meaningful activity, level of ward security, and therapeutic hold on the EssenCES significantly predicted QOL on a range of specific QOL domains. These variables accounted for 40% of the variance for total QOL score. Engagement in meaningful activity added the largest contribution to total QOL score accounting for 30% of the variance.

**Conclusions:**

This study shows that provision of meaningful activities, level of ward security and therapeutic hold may contribute to QOL amongst forensic mental health inpatients.

## Background

Quality of Life (QOL) is important in forensic mental health inpatient settings [1–6]. There are moral, ethical, clinical and legal reasons for considering QOL as an outcome [2]. Coid [2] argues that ‘any discussion on the QOL of a detained person must proceed on the principle that basic or moral rights exist... and that they should be reflected in the law’.

QOL has long been regarded as a core outcome for service users with a diagnosis of schizophrenia in general mental health settings [7–13]. For instance, numerous studies have found that higher levels of clinical symptoms are associated with reduced QOL for patients with schizophrenia [9–13]. In particular, many cross-sectional and longitudinal studies showed that lower depressive symptoms and a higher level of social functioning significantly predicted improved QOL [14]. Multivariate analyses explained 53% of the total variance in one such study where the significant predictors were depressive symptoms and social functioning [14]. Furthermore, a recent meta-analysis confirmed an association between neurocognition and expert reported QOL (i.e daily functioning), but indicated that there is largely no association between neurocognition and self reported QOL [15]. Similarly, recent studies have shown that negative symptoms are associated with expert-related QOL however there are no significant associations with self reported QOL. Moreover, it has been shown that psychosocial interventions are associated with improved QOL [16] and in particular, meaningful and satisfying daily activities were consistently associated with QOL [17, 18].

In recent years greater attention has been given to the concept of QOL in forensic mental health [1–6]. For instance, with the shift towards recovery-focused practice, QOL and wellbeing are now recognised as important outcomes [1, 3]. Drennan et al. [3], writing on recovery in forensic mental health suggested that from the perspective of service providers, QOL should be an essential outcome. Similarly, the U.K. College of Occupational Therapists [COT] has recommended in a key practice guideline that occupational therapists working on forensic mental health should consider the impact of interventions on QOL [19]. Moreover, the European Union Cooperation in Science and Technology [20] have recently set up an international forum for experts in the area of forensic mental health to gain and share knowledge. One of the primary goals of this forum is to develop a greater understanding of the factors that contribute to QOL for forensic inpatients [20].

Additionally, it is known that QOL is a positive protective factor in reducing both short term and long term criminal recidivism and therefore emphasis on this outcome contributes to public protection [21, 22]. A number of theories associate QOL with reduced recidivism. For example, the conjecture that risk of criminal offending reduces when individuals have a ‘good life’ is central to the Good Lives Model [23]. In addition, the relationship between social indicators of quality of life and recidivism has been shown in numerous studies. For example, the association between reduced recidivism and engagement in meaningful leisure activities, work and adequate management of finances has been demonstrated [21–27]. Because QOL and subjective wellbeing are associated with reducing recidivism, the concept of QOL is of central importance within forensic mental health.

The World Health Organisation has defined QOL as an ‘individual’s perception of their position in life in the context of culture and value systems in which they live and in relation to their goals, expectations, standards, and concerns’ [28]. Forensic mental health services provide treatment for people with mental illnesses such as schizophrenia who come into contact with law enforcement agencies as a consequence of their mental disorder, or who cannot be safely managed within another service. Admission to a forensic mental health hospital is almost always compulsory and via the courts. Length of stay in a forensic inpatient setting is often greater than 5 years [29–32]. In addition, many civil liberties may be restricted. Because forensic mental health service users are detained within restrictive settings, optimising QOL is an essential component of their care [1–3]. In addition the QOL experienced by forensic mental health service users may be lower than that experienced by service users in a general mental health population and also non- clinical populations [8, 9, 33–35]. This is because the clinical presentation of this patient group is complex [33–36]. For instance, treatment resistant psychopathology and impaired neurocognition are prevalent [33–36]. Because service users may have a longer period of stay within forensic inpatient services and a more complex presentation in comparison to their non-forensic counterparts, maximising their QOL is an essential treatment objective.

To our knowledge, only three studies have explored factors associated with QOL within a forensic mental health setting [37–39]. Factors such as level of ward security, diagnosis, use of atypical antipsychotics, and length of stay were considered in one such study by Long et al. [37]. The findings did not support an association between better QOL and length of hospital stay, a diagnosis of schizophrenia, or the use of atypical antipsychotics. However, this study did report that service users had a significantly higher level of satisfaction on wards with a lower level of security. The authors additionally reported that perceptions of personal control, mastery, freedom of choice and privacy are potential reasons for this association with better QOL. The study also showed an association between better QOL and lower levels of anxiety, depression and hostility. The authors concluded that interventions that enhance service users’ perceptions of control and mastery are important [37]. Conversely, Trizna & Adamowski [38] found that there was no significant difference between quality of life scores on medium and low secure wards. The authors highlighted that the differences between unmet needs on medium secure wards as compared to lower secure wards did not translate to improvements in self report quality of life or improved satisfaction with care. Interestingly within this study, service users on lower levels of security had increased psychopathology. The authors reported that service users had been in detention more frequently on lower secure wards hence had a more complex and severe psychopathology. Within this study increased psychopathology on lower security levels may explain the little change in self report QOL scores between wards. Swinton et al. [39] investigated QOL amongst service users with personality disorder and schizophrenia detained within a secure setting. Although environmental conditions and length of stay were similar for both groups the authors reported that service users with personality disorder subjectively reported poorer QOL than service users with schizophrenia. Thus far, research exploring factors associated with QOL in forensic mental health is sparse. Nevertheless findings from these studies indicate that level of ward security, psychopathology and diagnoses are associated with QOL for service users detained within a forensic mental health setting. Despite QOL and wellbeing now being recognised as core outcomes in forensic mental health there remains a paucity of research investigating the predictors of QOL in forensic mental health inpatient settings. Therefore, this study aims to analyse the predictors of QOL from a range of psychosocial factors that are relevant for forensic mental health service users. To our knowledge, no previous research has explored these issues.

### Objective/aim of study

We hypothesised that some or all of a range of psychosocial variables would account for a significant proportion of the measured variance in total QOL score and individual scores across specific QOL domains. The psychosocial variables included engagement in meaningful activity, ward atmosphere [therapeutic hold, experienced safety and patient cohesion], social and occupational functioning, community leave, length of stay and level of ward security.

## Methods

### Study design

This study is a naturalistic, cross-sectional, observational study.

### Setting

The national forensic mental health service (NFMHS) provides specialised care for adults who have a mental disorder and are at risk of harming others and themselves. Service users within the NFMHS are cared for in a safe and secure therapeutic hospital. Care is provided for those who are both residing within the hospital and within the community. Stratified care pathways via high, medium and low-secure wards within the hospital enable a safe therapeutic environment to maximise recovery potential.

### Participants

This study included male inpatients who were not in an acute stage of illness and who had a diagnosis of schizophrenia or schizoaffective disorder. Participants resided in high and medium secure rehabilitation wards and low secure wards. This study included a national cohort of forensic inpatients. Participants were aged between 20 and 65 years. All participants were diagnosed with schizophrenia or schizoaffective disorder by a consultant psychiatrist. Diagnoses were made in accordance with the Diagnostic and Statistical Manual of Mental Disorders IV-TR [40] using SCID-I based on interview and chart review.

### Variables

#### World Health Organisation QOL assessment (WHOQOL Bref)

The most widely used definition of QOL was provided by the World Health Organization Quality of Life (WHOQOL) Group in 1995. Hence, the most widely used scale for measuring QOL for service users with schizophrenia is the WHOQOL Bref [41]. This measure is deemed to adequately capture the salient QOL concepts for service users in a forensic setting and has been used to validate more specific forensic quality of life measures [5] and is used as an outcome measure in routine clinical practice at the research site. In addition the WHOQOL Bref is a valid, useful and reliable measure of QOL for people with schizophrenia [42, 43]. Psychometric properties were analysed using cross-sectional data obtained from a survey of adults carried out in 23 countries [*n* = 11,830]. The instrument demonstrated adequate internal consistency [Cronbach’s alpha coefficients were higher than 0.7 across the 4 domains and for the total score] and all items had acceptable correlation with the corresponding domain scores [*r* = 0.32–0.73]. It is regarded as a valid assessment of QOL, which is reflected by its four domains: physical, psychological, social and environment [42, 43]. Each individual domain is scaled in a positive direction. The total score on the four domains combined represents overall QOL and is scaled in a positive direction (i.e. higher scores denote higher QOL) with scores ranging between 0 and 100.

#### Essen climate evaluation schema (EssenCES)

The EssenCES is a short questionnaire, developed for assessing aspects of the social and therapeutic atmosphere of forensic psychiatric wards [44]. It has three dimensions: ‘patient cohesion and mutual support’ (PC; e.g. ‘service users care for each other’), ‘experienced safety’ (ES; e.g. ‘there are some really aggressive service users in this unit’) and ‘Therapeutic hold’ (e.g. ‘staff take a personal interest in the progress of service users’). Each question is scored on a scale between 0 and 4. The total score in each domain (patient cohesion, experienced safety, therapeutic hold) is scored positively on a scale between 0 and 20. Psychometric properties have been validated for UK forensic samples [44–46]. An examination of construct validity with the English EssenCES showed satisfactory internal consistency for all EssenCES scales, and using confirmatory factor analysis the expected three-factor structure was confirmed with residents in secure hospital settings [45].

#### Engagement in meaningful activity survey (EMAS)

The EMAS [47] assesses aspects of the meaning of activity with a particular emphasis on the synergy between an activity and an individual’s value system and needs. The EMAS is regarded as a valid and reliable tool and the instrument measures a unidimensional construct. It measures 12 facets of the meaningfulness of activities. Test-retest reliability and Cronbach Alpha are .69 and .84, respectively [47]. Scoring is conducted by summing the responses [ranging from 1 = Rarely to 4 = Always] of the 12 EMAS items for a possible score range of 12–48. The scale is scored positively and persons may be classified as perceiving the meaningfulness of their activities as being either low (EMAS < 29), moderate (EMAS 29–41) or high (EMAS > 41) [47].

#### Social and occupational functioning assessment scale (SOFAS)

The SOFAS is a global rating of current functioning which is scored positively on a scale from 0 to 100. Higher scores represent higher levels of functioning. The SOFAS has adequate psychometric properties [48]. A six-month cohort of general adult psychiatric inpatients were followed for up to two years to contrast the validity of *DSM-IV* measures of functioning—the Global Assessment of Functioning (GAF) and the Social and Occupational Functioning Assessment Scale (SOFAS) [48]. SOFAS and GAF scores on admission were found to significantly correlate with duration of hospital admission, and the SOFAS ratings on discharge were significantly correlated with psychiatric outcome at two years. The authors concluded that the SOFAS appeared to have better predictive validity and concurrent validity than the GAF and the SOFAS may be a more clinically useful measure of functioning [48, 49].

### Community leave status

Community leave status refers to whether a service user has access to the community or not. Based on risk assessment, selected service users will have access to the community for specified periods to engage with interventions that are intertwined with rehabilitation goals. Typically, service users who have progressed in the care pathway have community leave and are usually accompanied in the community by staff who work wihin the hospital. Risk factors are usually reduced and symptoms stabilised before community leave is recommended. Service users who have access to the community are usually well engaged with hospital rehabilitation programs. Access to the community is used for rehabilitation interventions such as building skills for using public transport and shopping, attending work or education programmes and participating in leisure activities such as going to the cinema, a museum or going to a coffee shop. For the purpose of this analysis service users without community leave were coded as zero and service users with community leave were coded as one.

### Length of stay

The length of stay is calculated from the date of admission to the date in which data were captured.

### Level of ward security

Service users were residing on high secure, medium secure, low secure wards or within a pre-discharge hostel. All wards are on the site of the Central Mental Hospital. The level of ward security was coded ordinally. The hostel setting (lowest level of security) was coded as one, the low secure rehabilitation ward was coded as two, the medium secure rehabilitation ward was coded as three, the medium secure sub-acute ward was coded as four, and the high secure ward was coded as five.

### Data sources/measurement

Participants were invited to participate within the study and each participant completed questionnaires which measured QOL, ward atmosphere, and engagement in meaningful activity. Data on occupational functioning were measured by clinicians who best knew the service users level of functioning. Length of stay and community leave status were also collected on the same day by lead author and co-authors. Participants were provided with questionnaires by clinicians who were not working directly with service users. The data collection tools used were the World Health Organisation QOL Assessment (WHOQOL- Bref) to measure QOL, the ‘Essen Climate Evaluation Schema’ (EssenCES) to measure ward atmosphere, the Social and Occupational Functioning scale (SOFAS) and the Engagement in Meaningful Activity Survey (EMAS). There were other variables considered such as community leave status, length of stay and level of ward security.

### Bias

We considered a national cohort of forensic mental health patients so as to minimise selection bias.

### Study size

Eligible participants for this study were recruited from the inpatient population residing in high, medium or low-secure wards (*n* = 67). Of the 67 that were asked if they wanted to participate in the study, 52 (78%) consented and completed all questionnaires. There were 7 service users who originally consented but did not complete all of the questionnaires and withdrew from the study and there were 8 people who did not give consent.

### Statistical methods

Data were analysed using Statistical Package for the Social Sciences (SPSS) version 23.

A stepwise multiple regression analysis was conducted to determine the unique contributions of clinical variables to QOL (WHOQOL-Bref total score). In addition a stepwise multiple regression analysis was used to determine the unique contributions of clinical variables to the specific domains within the WHOQOL Bref (physical health, psychological health, social relationships and environment). Due to the range of variables considered within the analysis it was deemed appropriate to use this method in identifying significant predictors of QOL. In the regression models the dependent variable was QOL as measured by the total score of the WHOQOL Bref across all domains and total score on individual domains. Independent variables were engagement in meaningful activity as measured by the EMAS, social and occupational functioning as measured by the SOFAS, ward atmosphere (therapeutic hold, experienced safety, patient cohesion) as measured by the EssenCES, level of ward security, community leave status and length of stay.

### Ethics, consent and permissions

The study was approved by the Research, Ethics and Audit Committee of the National Forensic Mental Health Service (NFMHS). All participants gave informed signed consent. An information sheet was provided to each participant. The information sheet detailed the aim of the study, the potential risks and benefits and the reassurance that participation was voluntary and that participation could be withdrawn at any time. This study was compliant with the Declaration of Helsinki.

## Results

### Participants

Of the 67 that were asked if they wanted to participate in the study 52 (78%) consented and completed all questionnaires. There were 7 service users who originally consented but did not complete all of the questionnaires and withdrew from the study and there were 8 people who did not give consent.

### Descriptive data

The median length of stay was 5.5 yrs. (SD 6.8) with a minimum of 0.5 yrs. and a maximum of 35.5 yrs. The mean age of participants was 40.3 yrs. (SD 16.4). Participants resided within high security [*n* = 6], medium security (*n* = 14), medium security rehabilitation (*n* = 15), low security rehabilitation ward (*n* = 11) and low security hostel (n = 6). Community leave status was dichotomised between those with no community leave (*n* = 16) or those with community leave (*n* = 36). There were 67 service users who met inclusion criteria. All service users were male and met diagnostic criteria for schizophrenia or schizoaffective disorder (*n* = 67). 8 service users declined to participate. 7 service users did not to complete all questionnaires and withdrew from the study. Data were therefore available for 52 participants which are presented in Table 1.

**Table 1:** Describes demographic and clinical characteristics *n* = 52

Patient characteristics				
*Mean age*	Years 40.3	SD 16.4	Min 27.0	Max 55.0
*Median length of stay*	Years 5.5	SD 6.8	Min 0.5 yrs	Max 33.5 yrs
*Ward location*				
Low secure hostel	6 [12%]			
Low secure rehabilitation ward	11 [20%]			
Medium security rehabilitation	15 [29%]			
Medium security sub-acute	14 [27%]			
High secure [long stay]	6 [12%]			
*Community leave status*				
Community leave	36 [70%]			
No community Leave	16 [30%]			
*Primary Diagnosis*				
Schizophrenia/ Schizoaffective disorder	52 [100%]			

### Outcome data

The mean WHOQOL Bref total QOL score and WHOQOL Bref individual domain scores (physical health, psychological health, social relationships and environment) are detailed in Table 2. In addition the mean score for the engagement in meaningful activity scale, social and occupational functioning scale and EssenCES constructs (therapeutic hold, patient cohesion and experienced safety) are also detailed.

**Table 2:** Describes mean and standard deviations of variables [n = 52]

	*Mean*	*Std. Deviation*
*WHOQOL Bref [QOL] Total*	*66.0*	*12.9*
*WHOQOL Bref Physical Health*	*73.8*	*12.9*
*WHOQOL Bref Psychological Health*	*64.6*	*12.6*
*WHOQOL Bref Social Relationships*	*57.6*	*19.3*
*WHOQOL Bref Environment*	*67.9*	*16.4*
*Engagement in meaningful activity scale [EMAS]*	*35.4*	*8.2*
*Social and occupational functioning scale [SOFAS]*	*56.2*	*12.5*
*EssenCES Therapeutic Hold*	*13.7*	*4.3*
*EssenCES Patient Cohesion*	*12.1*	*4.3*
*EssenCES Experienced safety*	*16.3*	*3.4*

**Table 3:** Shows stepwise regression model for total WHOQOL Bref score with significant variables entered

*Model 1*	*Unstandardized coefficients*		*Standardized coefficients*	*t*	*Sig.*
	*B*	*Std. Error*	*Beta*		
*1] Constant*	*34.99*	*6.70*		*5.22*	*p < 0.0001*
*Engagement In Meaningful Activity Scale*	*0.874*	*0.185*	*0.557*	*4.738*	*p < 0.0001*
*Model 2*	*Unstandardized coefficients*		*Standardized coefficients*	*t*	*Sig*
	*B*	*Std. Error*	*Beta*		
*2] Constant*	*53.65*	*10.90*		*4.927*	*p < 0.0001*
*Engagement in meaningful activity survey*	*0.767*	*0.185*	*0.489*	*4.144*	*p < 0.0001*
*Level of ward security*	*−2.33*	*1.092*	*−0.251*	*−2.133*	*p = 0.038*
*Model 3*	*Unstandardized coefficients*		*Standardized coefficients*	*t*	*Sig.*
	*B*	*Std. Error*	*Beta*		
*3] Constant*	*52.99*	*10.46*		*5.066*	*p < 0.0001*
*Engagement in meaningful activity survey*	*0.478*	*0.219*	*0.305*	*2.185*	*p = 0.034*
*Level of ward security*	*−2.551*	*1.053*	*−0.275*	*−2.422*	*p = 0.019*
*EssenCES Therapeutic Hold*	*0.902*	*0.398*	*0.305*	*2.264*	*p = 0.028*

**Table 4:** Shows stepwise regression model with significant predictors of the WHOQOL Bref physical domain detailed

*Model 1*	*Unstandardized coefficients*		*Standardized coefficients*	*t*	*Sig.*
	*B*	*Std. Error*	*Beta*		
*1] Constant*	*60.94*	*5.73*		*10.64*	*p < 0.0001*
*EssenCES therapeutic hold*	*0.945*	*0.400*	*0.317*	*2.36*	*p = 0.022*

**Table 5:** Shows stepwise regression model with significant predictors of the WHOQOL Bref psychological health domain detailed

*Model 1*	*Unstandardized coefficients*		*Standardized coefficients*	*t*	*Sig.*
	*B*	*Std. Error*	*Beta*		
*1] Constant*	*26.16*	*9.54*		*2.74*	*p = 0.008*
*Engagement In Meaningful Activity Scale*	*1.08*	*0.262*	*0.505*	*4.133*	*p < 0.0001*
*Model 2*	*Unstandardized Coefficients*		*Standardized Coefficients*	*t*	*Sig*
	*B*	*Std. Error*	*Beta*		
*2] Constant*	*54.08*	*15.40*		*3.51*	*p = 0.001*
*Engagement In Meaningful Activity Scale*	*0.924*	*0.262*	*0.430*	*3.53*	*p = 0.001*
*Level of ward security*	*−3.49*	*1.54*	*−0.275*	*−2.26*	*P = 0.029*

**Table 6:** Shows stepwise regression model with significant predictors of the WHOQOL Bref social relationships domain detailed

*Model 1*	*Unstandardized coefficients*		*Standardized coefficients*	*t*	*Sig.*
	*B*	*Std. Error*	*Beta*		
*1] Constant*	*26.50*	*11.26*		*2.35*	*p = 0.023*
*Engagement in Meaningful Activity Scale*	*0.877*	*0.310*	*0.372*	*2.83*	*p = 0.007*

**Table 7:** Shows stepwise regression model with significant predictors of the WHOQOL Bref environment domain detailed

*Model 1*	*Unstandardized coefficients*		*Standardized coefficients*	*t*	*Sig.*
	*B*	*Std. Error*	*Beta*		
*1] Constant*	*39.80*	*6.40*		*6.22*	*p < 0.001*
*EssenCES Therapeutic Hold*	*2.06*	*0.447*	*0.546*	*4.60*	*p < 0.0001*
*Model 2*	*Unstandardized coefficients*		*Standardized coefficients*	*t*	*Sig*
	*B*	*Std. Error*	*Beta*		
*2] Constant*	*64.15*	*10.76*		*5.95*	*p > 0.001*
*EssenCES Therapeutic Hold*	*0.924*	*0.262*	*0.430*	*3.53*	*p = 0.001*
*Level of ward security*	*−3.60*	*1.32*	*−0.305*	*−2.73*	*P = 0.009*

### Main results

Table 3 shows results of stepwise regression analysis for the overall model. Within this model, engagement in meaningful activity, level of ward security and therapeutic hold statistically predicted total QOL score across all domains. Engagement in meaningful activity was automatically entered first into the overall model *R*^2^ = 0.31, *F* (1, 51) = 22.5, *p* < 0.0001, adjusted *R*^2^ = 0.30 and added the largest contribution. Level of ward security was automatically entered second into the overall model *R*^2^ = 0.37, *F* (2, 50) =14.3, *p* < 0.0001, adjusted *R*^2^ = 0.34 and finally EssenCES therapeutic hold was entered into the overall model *R*^2^ = 0.43, *F* (3, 49) =12.0, *p* < 0.0001, adjusted *R*^2^ = 0.40. The other variables included in the model did not add significantly to the prediction and were automatically removed. The overall model of engagement in meaningful activity, level of ward security, and therapeutic hold was statistically significant *R*^2^ = 0.43, *F* (3, 49) =12.0, *p* < 0.0005; adjusted *R*^2^ = 0.40.

Table 4 shows results of stepwise regression model with significant predictors of the WHOQOL Bref ‘physical health’ domain detailed. Within this model EssenCES therapeutic hold alone predicted WHOQOL Bref ‘physical health’ R^2^ = 0.08, F (1, 51) = 5.6, *p* = 0.02. The other variables included in the model did not add significantly to the prediction and were automatically removed.

Table 5 shows results of stepwise regression model with significant predictors of the WHOQOL Bref ‘psychological health’ domain. Engagement in meaningful activity was automatically entered first into this model R^2^ = 0.24, F (1, 51) = 17.1, *p* < 0.001 and level of ward security was entered second R^2^ = 0.30, F (2, 50) = 11.8, *p* < 0.001. The other variables included in the model did not add significantly to the prediction and were automatically removed.

Table 6 shows results of stepwise regression model with significant predictors of the WHOQOL Bref ‘social relationships’ domain. Engagement in meaningful activity was automatically entered into this model R^2^ = 0.12, F (1, 51) = 8.0, *p* = 0.007. The other variables included in the model did not add significantly to the prediction and were automatically removed.

Table 7 shows results of stepwise regression model with significant predictors of the WHOQOL Bref ‘environment’ domain. EssenCES therapeutic hold was automatically entered first into this model R^2^ = 0.28, F (1, 51) = 21.2, *p* < 0.001 and level of ward security was entered second R^2^ = 0.37, F (2, 50) = 15.7, *p* < 0.001. The other variables included in the model did not add significantly to the prediction and were automatically removed.

## Discussion

### Key results

We hypothesized that some or all of a range of psychosocial variables would account for a significant proportion of the measured variance in QOL. The psychosocial variables included engagement in meaningful activity, ward atmosphere (therapeutic hold, experienced safety and patient cohesion), social and occupational functioning, community leave, length of stay and level of ward security. Engagement in meaningful activity, level of ward security, and therapeutic hold all significantly accounted for a proportion of the variance for QOL. The three variables together accounted for 40% of the variance of total QOL score across all domains. In addition within specific QOL domains, EssenCES therapeutic hold significantly accounted for a small proportion of the variance (8%) in the ‘physical health’ domain. Engagement in meaningful activity and level of ward security together significantly accounted for a large proportion of variance (30%) within the ‘psychological health’ domain. Engagement in meaningful activity significantly accounted for 12% of the variance in the ‘social relationships’ QOL domain and finally EssenCES therapeutic hold and level of ward security together contributed to a large proportion of the variance (37%) within the QOL ‘environment’ domain. Length of stay, social and occupational functioning, patient cohesion, experienced safety [EssenCES] and community leave did not make a contribution to the model in this study. This study contributes to an understanding of the predictors of QOL for service users within a forensic hospital.

### Interpretation

#### Meaningful activity and QOL

This study shows a positive association between engagement in meaningful activity and QOL for service users who reside within a forensic mental health hospital. In particular engagement in meaningful activity made a significant contribution to the QOL ‘psychological health domain and QOL ‘social relationships domain’. The association between engagement in meaningful activity and wellbeing is a well known tenet of occupational therapy theory [50–54]. This present study provides empirical support for this relationship in a forensic mental health inpatient setting. The provision of meaningful activities for service users within this setting is a key recommendation made within the college of occupational therapists ‘practice guidelines for forensic mental health’ and the ‘quality network for forensic mental health standards’ [19, 54]. The results of this study provide further support for these recommendations. It has previously been shown that meaningful activity may provide purpose, structure, social connectnedness, routine and pleasure and these factors may contribute to a sense of personal agency and wellbeing [50–54]. Furthermore, the skills and competence developed as a result of participating in meaningful activity may improve functioning and subsequently provide a more seamless reintegration into the community upon discharge. Moreover, engagement in meaningful activity may reduce the person’s risk for mental illness relapse. In addition engagement in meaningful activity may prevent readmission to hospital and contact with the criminal justice system [21–27, 55].

#### Level of ward security and QOL

This present study shows a significant negative association between level of ward security and QOL. In particular, level of ward security makes a significant contribution to the QOL ‘psychological health’ domain and QOL ‘environment’ domain. Similarly, Long et al. [37] showed that higher levels of security were associated with a lower QOL. The authors additionally reported that perceptions of personal control, mastery, freedom of choice and privacy are potential reasons for this association with better QOL [37]. This present study shows that service users on lower secure wards have increased QOL scores. The particular characteristics of lower secure wards within this setting include increased autonomy, privacy and personal control. In addition, oppurtunities to manage activities of daily living are optimised. For instance, service users can manage their own self catering, shopping, personal care and deploy community living skills regularly as many have access to the community. Additionally increased access to a variety of leisure oppurtunities, vocational projects, education or work, and a greater choice regarding personal routines are other notable differences. An array of factors within the physical, social and therapeutic environment that stem from physical, procedural and relational security hospital policies on lower secure wards may additionally explain associations with better QOL scores. For instance, increased control over one’s personal routine and increased privacy may be specific factors that contribute to QOL. Furthermore, there may be other factors which account for this association. Trizna and Adamowski [38] showed that there was no significant difference in QOL between medium and lower secure wards. Within their study psychopathology was increased in those at lower levels of security [38]. Conversely on the site where research data was collected for this study service users on lower levels of security generally have decreased pscyhopathology. A decrease in symptom severity has previously been shown to be associated with improved subjective QOL [9–13]. Thus, there maybe a range of factors that account for this relationship.

#### Therapeutic relationship and QOL

Additionally, the results of this study show a positive association between therapeutic relationship and QOL. In particular therapeutic hold on the EssenCES significantly contributed to QOL within the ‘physical health’ domain and ‘environment’ domain. The results provide further evidence to support the importance of optimising therapeutic relationships. It is well documented in the recovery literature [1, 3, 56] that an optimisation of therapeutic rapport can be achieved primarily through avoiding coercive choices, involving individuals in care planning/service development, identifying and working towards individualised goals and providing a safe and non-judgemental environment. Additionally, a positive therapeutic relationship may reduce symptomatology, increasing engagement with services and medication adherence [56–59]. Hence optimising therapeutic relationships may improve QOL in this setting.

#### Challenges in secure services

There are multifaceted challenges to providing activities that are meaningful, optimising therapeutic rapport and positive risk taking within forensic inpatient settings [55, 60, 61]. The confined environment of a secure inpatient ward with limited access to the community may present fewer opportunities for engaging in meaningful activities, practicing and applying skills, and providing choice and autonomy which may impact on QOL [55, 60, 61]. Additionally, there can be tension between safety, security and the provision of a therapeutic environment. For instance, features such as secure physical and social environments are required however these features can also result in restricted routines, autonomy and choice which can negatively affect therapeutic relationships [55]. Furthermore the clinical complexities may account for deficits in occupational performance [36]. For instance, neurocognitive deficits associated with schizophrenia contribute to impaired abilities to perform instrumental activities of daily living and engage with meaningful activities [36]. Conversely if abilities to perform instrumental activities of daily living and meaningful activities are intact, opportunities to deploy skills may be limited due to risk management [60–62]. Consequently, optimising the environment to afford service users an opportunity to deploy and retain skills whilst remaining as inpatients is an added complexity within a forensic mental health inpatient environment.

#### Opportunities to make progress

Optimising ward routines by facilitating autonomy and independence within the context of positive risk taking should be prioritised. However it must be acknowledged that there may be a heterogeneous group of service users with a range of security needs/risk issues residing on a single medium security ward. Hence, adjustments to the ward physical layout, therapeutic and ward operational policies may be necessary to stratify activity provision effectively, to ensure the least restrictive environment and to optimise therapeutic relationships.

In addition, service user participation in the design and delivery of therapeutic programmes should be considered [3, 55]. For instance, this could include participation in planning/governance committees and in the organisation of ward and personal activity timetables. Ward-based forums or community meetings for service users and staff to discuss daily life on a ward and the experiences of those that participate in it may be useful, particularly in making adjustments at a ward operational level. In addition, instilling the recovery principles particularly co-production may enhance therapeutic rapport and improve the quality of service provision.

Finally, it is important that clinicians explore potential factors impacting on ‘meaning’ within this setting. For instance, meaningful occupation is thought to reflect one’s personal values, beliefs, goals, interests, world-view and experiences including pleasure, social connectedness, competency, control and enjoyment [47, 56–59]. Exploration of self identity could be considered at a number of stages throughout the care pathway. Occupational therapists could utilise theories within occupational science to support and guide practice within this area.

#### Limitations

This study was conducted within a secure forensic setting which may limit the generalisability of the findings to non-forensic settings. In addition the sample size was small with 52 service users participating. Moreover, social desirability, emotional state, psychopathology, neurocognition, insight or co-morbidities were not controlled for at the time of QOL evaluation. These factors may confound the measurement of QOL. There are also limitations with the cross sectional design. This means that the results only show associations and not a direct causal relationship. Repeating the QOL measure on a number of occasions within a prospective design would be beneficial to account for confounding factors.

The use of a self report QOL measure alone has specific limitations. Becker et al. [63] reported that QOL assessments should be carried out not only via the patient, but also via professional helpers and key informants. However the aim of this study was to analyse self- report QOL and Voruganti et al. [64] reported that service users with schizophrenia, while in remission and willing to cooperate, are capable of adequate estimation of their QOL. Future research should consider asking staff members to rate QOL using a proxy measure to get both perspectives.

The present study however combined objective and subjective measures in the model. Level of ward security is an objective measure and did influence the subjective rating of QOL. It is interesting therefore that level of community leave dropped out of this statistical model – possibly because it is confounded with level of ward security. Length of stay however is also an objective measure and did not relate to QOL. Finally, this is a cross-sectional study, so patients who have shorter periods of stay may be underrepresented.

### Generalisability

This study was conducted with service users with a diagnosis of schizophrenia and schizoaffective disorder within a secure forensic hospital across a care pathway stratified by levels of security. The results of this study may be generalisable to other forensic mental health settings with a similar model of care.

## Conclusion

The purpose of this study was to develop a greater understanding of the predictors of QOL within an inpatient forensic mental health setting. The results show that there is an association between meaningful activity, level of ward security, therapeutic hold and domains of QOL. The results provide support for a central assumption of occupational therapy theory; that there is a relationship between engagement in meaningful activity and wellbeing/QOL. In addition the results provide support for the least restrictive principle and theories about the importance of therapeutic relationships. Forensic service providers should routinely measure QOL. Furthermore, ensuring the least restrictive environment and optimising therapeutic relationships should be a priority. Occupational therapists are excellently placed and skilled to pioneer initiatives that enrich inpatient forensic environments with meaningful activities. It is anticipated by the authors that the QOL of service users who reside as inpatients within secure environments for many years may be improved by increasing access to meaningful activities, positive risk taking and optimisation of therapeutic relationships. Future studies investigating the effects of occupational therapy interventions on outcomes such as QOL and occupational performance/functioning are needed.

## Abbreviations

EMAS: Engagement in Meaningful Activity Survey
ESSENCES: Essen Climate Evaluation Schema
QOL: Quality of life
SOFAS: Social and Occupational Functioning Scale
WHOQOL Bref: World Health Organisation QOL Bref

## References

[1] Mann B, Matias E, Allen J (2014). Recovery in forensic services: facing the challenge. Adv Psychiatr Treat.

[2] Coid JW (1993). QOL for patients detained in hospital. Brit J Psychiat.

[3] Drennan G, Wooldridge J (2013). Making recovery a reality in forensic settings. ImROC briefing paper.

[4] Van Nieuwenhuizen CH, Schene MH & Koeter M. QOL in forensic psychiatry: an unreclaimed territory?, Int Rev Psychiatr. 2002; 14:3: 198–202.

[5] Vorstenbosch E, Bouman Y, Braun P (2014). Psychometric properties of the forensic inpatient QOL questionnaire: QOL assessment for long-term forensic psychiatric care. Health Psychol Behav Med.

[6] Mulholland F, Rooney B, Pettigrew J (2015). The hen project: introducing vocational opportunity into a high secure forensic unit. Irish J Occup Therapy.

[7] Lehman AF, Ward NC, Linn LS (1982). Chronic mental patients: the QOL issue. Am J Psychiat..

[8] Skantze K, Malm U, Denker SJ (1992). Comparison of QOL with standard of living in schizophrenic out-patients. Brit J Psychiat..

[9] Atkinson M, Zibin SH, Chuang H (1997). Characterizing QOL among patients with chronic mental illness: a critical examination of the self-report methodology. Am J Psychiat.

[10] Eack S, Newhill C (2007). Psychiatric symptoms and quality of life in schizophrenia: a meta-analysis. Schizophr Bull.

[11] Vatne S, Bjorkly S (2008). Empirical evidence for using subjective quality of life as an outcome variable in clinical studies. A meta-analysis of correlates and predictors in persons with a major mental disorder living in the community. Clin Psychol Rev.

[12] Priebe S, McCabe R, Junghan U (2011). Association between symptoms and quality of life in patients with schizophrenia: a pooled analysis of changes over time. Schizophr Res.

[13] Galuppi A, Turola MC, Nanni MG, Mazzoni P, Grassi L (2010). Schizophrenia and quality of life: how important are symptoms and functioning?. Int J Ment Health Syst..

[14] Meesters PD, Comijs HC, de Haan L (2011). Subjective quality of life and its determinants in a catchment area based population of elderly schizophrenia patients. Schizophr Res.

[15] Tolman AW, Kurtz MM (2012). Neurocognitive predictors of objective and subjective quality of life in individuals with schizophrenia: a meta-analytic investigation. Schizophr Bull.

[16] Pitkanen A, Valimaki M, Kuosmanen L (2012). Patient education methods to support quality of life and functional ability among patients with schizophrenia: a randomised clinical trial. Qual Life Res.

[17] Eklund M (2009). Work status, daily activities and quality of life among people with severe mental illness. Qual Life Res.

[18] Goldberg B, Brintnell S, Goldberg J (2002). The relationship between engagement in meaningful activities and quality of life in persons disabled by mental illness. Occup Ther Ment Health.

[19] College of Occupational Therapists. Occupational therapists’ use of occupation-focused practice in secure hospitals: practice guideline. 2012. London: COT.

[20] Braun P. Towards an EU research framework on forensic psychiatric care. 2013.http://www.cost.eu/COST_Actions/isch/IS1302. Accessed 4th January 2018.

[21] Bouman Y, Schene AH. Subjective well-being and recidivism in forensic psychiatric outpatients. Int J Forensic Ment Health. 2009;8(4)

[22] Abidin Z, Davoren M, Naughton L (2013). Susceptibility [risk and protective] factors for in-patient violence and self-harm. BMC Psychiatry..

[23] Ward T, Brown M (2004). The good lives model and conceptual issues in offender rehabilitation. Psychol Crime & Law..

[24] Bouman Y, Ruiter C, Schene AH (2010). Social ties and short-term self-reported delinquent behaviour of forensic personality disordered outpatients. Legal Criminol Psychol.

[25] Monahan J, Steadman HJ, Silver E, Appelbaum PS, Robbins PC, Mulvey E (2001). Rethinking risk assessment: the MacArthur study of mental disorder and violence.

[26] Ogloff JRP, Davis MR (2004). Advances in offender assessment and rehabilitation: contributions of the risk-need-responsivity approach. Psychol Crime & Law.

[27] Webster CD, Martin M, Brink J, Nicholls TL, Middleton C (2004). Short term assessment of risk and treatability [START]. Clinical guide for evaluation risk and recovery.

[28] Saxena S, Orley J (1997). WHOQOL Group. QOL assessment: The world health organization perspective. Eur Psychiatry.

[29] Andreasson H, Nyman M, Krona H (2014). Predictors of length of stay in forensic psychiatry: the influence of perceived risk of violence. Int J Law Psychiatry.

[30] Sharma A, Dunn W, O'Toole C (2015). The virtual institution: cross-sectional length of stay in general adult and forensic psychiatry beds. Int J Ment Health Syst.

[31] Davoren M, Byrne O, O'Connell P (2015). Factors affecting length of stay in forensic hospital setting: need for therapeutic security and course of admission. BMC Psychiatry.

[32] Shah A, Waldron G, Boast N (2011). Factors associated with length of admission at a medium secure forensic psychiatric unit. J Forens Psychiatry Psychol..

[33] Katschnig H, Freeman H, Sartorius N (2006). QOL in mental disorders.

[34] Barry MM, Crosby C (1996). QOL as an evaluative measure in assessing the impact of community care on people with long-term psychiatric disorders. Br J Psychiatry.

[35] Samuli I, Saarni S, Jonna P (2010). QOL of people with schizophrenia, bipolar disorder and other psychotic disorders. Br J Psychiatry.

[36] Reilly K, G O’ D, Sullivan D (2016). Study protocol: a randomised controlled trial of cognitive remediation for a national cohort of forensic mental health service users with schizophrenia or schizoaffective disorder. BMC Psychiatry.

[37] Long CG, McLean A, Boothby AJ (2008). Factors associated with QOL in a cohort of forensic psychiatric in-patients. Br J Forensic Pract.

[38] Trizna M, Adamowski T (2016). Assessment of needs and clinical parameters in forensic patients in low and medium security wards. Archives Psychiatry and Psychother.

[39] Swinton M, Oliver J, Carlisle J (1999). Measuring QOL in secure care: comparison of mentally ill and personality disordered patients. Int J Soc Psychiatary.

[40] American Psychiatric Association. Diagnostic and statistical manual of mental disorders 4th edition, Text Revision. Washington, DC, American Psychiatric Association; 2000.

[41] Karow A, Wittman L, Schöttle D, Schäfer I, Lambert M (2014). The assessment of quality of life in clinical practice in patients with schizophrenia. Dialogues Clin Neurosci.

[42] The WHOQOL Group (1998). The World Health Organization QOL assessment [WHOQOL]: development and general psychometric properties. Soc Sci Med.

[43] The WHOQOL Group (1998). Development of the World Health Organization WHOQOL-BREF QOL assessment. Psychol Med.

[44] Schalast N, Redies M, Collins M (2008). EssenCES, a short questionnaire for assessing the social climate of forensic psychiatric wards. Crim Behav Ment Health.

[45] Tonkin M, Howells K, Ferguson E (2012). Lost in translation? Psychometric properties and construct validity of the English Essen climate evaluation schema [EssenCES] social climate questionnaire. Eur J Psychol Assess.

[46] Dickens GL, Suesse M, Snyman P (2014). Associations between ward climate and patient characteristics in a secure forensic mental health service. J Forens Psychiatry Psychol.

[47] Eakman AM (2012). Measurement Characteristics of the Engagement in Meaningful Activities Survey in an Age-Diverse Sample. Am J Occup Ther.

[48] Rybarczyk B, Kreutzer J, Deluca J, Caplan B (2011). Social and occupational functioning assessment scale [SOFAS]. LXIII, encyclopedia of clinical neuropsychology.

[49] Hay P, Katsikitis M, Begg J (2003). A two-year follow-up study and prospective evaluation of the DSM-IV Axis V. Psychiat Ser.

[50] Law M, Sandy S, Leclair L. Occupation, health and well-being. Can J Occup Ther. 1998;65(2)

[51] Wilcock AAA (1993). Theory of the human need for occupation. J Occup Sci.

[52] Yerxa EJ, Baum S (1986). Engagement in daily occupations and life satisfaction among people with spinal cord injuries. Occup Ther J Res.

[53] Jackson J, Carlson M, Mandel D (1998). Occupation in lifestyle redesign: the well elderly study occupational therapy program. Am J OccupTher.

[54] Canadian Association of Occupational Therapists (1997). Enabling occupation: an occupational therapy perspective.

[55] Quality network for forensic mental health: Standards for medium secure services. Royal College of Psychiatrists Centre for Quality Improvement; 2014.

[56] Perkes D, Whiteford G, Charlesworth G (2015). Occupation- focussed practice in justice health and forensic mental health: using a practice-based enquiry approach. World Federation Occup Therapists Bulletin.

[57] Repper J, Ford R, Cooke A (1994). How can nurses build trusting relationships with people who have severe and long term mental health problems? Experiences of case managers and their clients. J Adv Nurs.

[58] Bjorkman T, Hansson L. Predictors of improvements in QOL of long term mentally ill individuals receiving care management. Eur Psychiatry. 2002;17 10.1016/s0924-9338(02)00621-1 11918991

[59] Weiss K, Smith T, Hull J (2002). Predictors of risk of non-adherence in outpatients with schizophrenia and other psychotic disorders. Schizophr Bull.

[60] Cleary M (2003). The challenges of mental health care reform for contemporary mental health nursing practice: relationship, power and control. Int J Ment Health Nurs.

[61] Farnworth L, Nikitin L, Fossey E (2004). Being in a secure forensic psychiatric unit: every day is the same, killing time or making the most of it. Br J Occup Ther.

[62] O’Connell M, Farnworth L (2007). Occupational therapy in forensic psychiatry: a review of the literature and a call for a united and international response. Br J Occup Ther.

[63] Becker A, Hagenberg N, Roessner V, et al. Evaluation of the self-reported SDQ in a clinical setting: do self-reports tell us more than ratings by adult informants? Eur Child Adolesc Psychiatry 2004;13: 17–24. 10.1007/s00787-004-2004-4 15243782

[64] Voruganti L, Heslegrave R, Awad AG, Seeman MV (1998). QOL measurement in schizophrenia: reconciling the quest for subjectivity with the question of reliability. Psychol Med.

